# Nutrient supply alters transcriptome regulation in adipose tissue of pre-weaning Holstein calves

**DOI:** 10.1371/journal.pone.0201929

**Published:** 2018-08-06

**Authors:** Leonel N. Leal, Josue M. Romao, Guido J. Hooiveld, Fernando Soberon, Harma Berends, Mark V. Boekshoten, Michael E. Van Amburgh, Javier Martín-Tereso, Michael A. Steele

**Affiliations:** 1 Trouw Nutrition R&D, Amersfoort, Utrecht, the Netherlands; 2 Department of Agricultural, Food and Nutritional Science, University of Alberta, Edmonton, Alberta, Canada; 3 Nutrition, Metabolomics and Genomics group, Division of Human Nutrition, Wageningen University, Wageningen, Gelderland, the Netherlands; 4 Trouw Nutrition USA, Highland, Illinois, United States of America; 5 Department of Animal Science, Cornell University, Ithaca, New York, United States of America; University of Illinois, UNITED STATES

## Abstract

Performance of dairy cows can be influenced by early life nutrient supply. Adipose tissue is diet sensitive and an important component in that process as it is involved in the regulation of energetic, reproductive and immunological functions. However, it is not clear how early life nutrition alters the molecular regulation of adipose tissue in calves and potentially adult individuals. This study aimed at determining how differences in pre-weaning nutrient supply alter gene expression profiles and physiology in omental adipose tissue. A total of 12 female Holstein calves were fed two levels of milk replacer supply: a restricted amount of 11.72 MJ of metabolizable energy (ME) intake per day (n = 6) or an enhanced amount of 1.26 MJ ME intake per kg of metabolic body weight (BW^0.75^), resulting in supply from 17.58 to 35.17 MJ ME intake per day (n = 6). All calves had ad libitum access to a commercial calf starter and water. Analysis of the transcriptome profiles at 54 ± 2 days of age revealed that a total of 396 out of 19,968 genes were differentially expressed (DE) between groups (p < 0.001, FDR < 0.1). The directional expression of DE genes through Ingenuity Pathway Analysis showed that an enhanced nutrient supply alters adipose tissue physiology of pre-weaned calves. Several biological functions were increased (Z-score > +2), including Lipid Metabolism (Fatty Acid Metabolism), Cell Cycle (Entry into Interphase, Interphase, Mitosis and Cell Cycle Progression), Cellular Assembly and Organization (Cytoskeleton Formation and Cytoplasm Development) and Molecular Transport (Transport of Carboxylic Acid). These changes were potentially orchestrated by the activation/inhibition of 17 upstream regulators genes. Our findings indicate that adipose tissue of calves under an enhanced nutrient supply is physiologically distinct from restricted calves due to an increased development/expansion rate and also a higher metabolic activity through increased fatty acid metabolism.

## Introduction

Early life is a critical period in which environmental factors such as nutrition can induce developmental plasticity towards altered biological functions in adulthood. Any event that occurs during this period can permanently influence the physiology of organisms: a process termed ‘programming’ or more recently ‘imprinting’[1]. The concept of early-life nutrition impacting long-term physiological responses in medical research has been well-established but only recently investigated in ruminants [2]. Studies on dairy calves have shown that pre-weaning intake, and thus gain, are positively correlated with lifetime milk production [3,4]. On the other hand, there is also evidence that excessive feeding can be detrimental to animal performance [5,6].

It is important to consider that dairy calves are traditionally restricted in milk or milk-replacer supply to approximately 10% of bodyweight as milk, which represents less than half of their voluntary consumption [7,8,9]. This contrasts with human and rodent early-life studies that typically investigate effects of excessive calorie consumption. More recently, calf studies have focussed on characterizing how milk supply influences metabolism and in particular insulin sensitivity [10,11]. So far, only one study investigated the epigenetic effects of pre-weaning nutrient supply in dairy calves, in which it was found that increased milk feeding induced the expression of genes and transcription factors associated with growth and development in rumen tissue [12].

Adipose tissue is critical in animal development as it participates in several biological processes, including energy balance, endocrine, thermogenic, reproductive, and immune functions [13,14,15]. It has been shown that its molecular regulation and physiology is responsive to diet in adult animals [16,17]. However, it is not clear how early life nutrition affects adipose tissue physiology and how early life adipose tissue modulation can impact the productive and reproductive performance of dairy cows.

Transcriptomic studies of key metabolic tissues offer a unique opportunity to understand how dietary manipulations can affect the physiology and development of farm animals by revealing a comprehensive view of the cellular and molecular regulation at a transcriptome level. We hypothesized that nutrient supply alters adipose tissue development and function in pre-weaning calves. Therefore, the objective of the present study was to determine how lifting the traditional nutrient supply in pre-weaning calves affects adipose tissue transcriptome expression and physiology.

## Materials and methods

### Experimental design, feeding and housing

The experimental protocols describing the management and animal care have previously been reported [18]. They were reviewed and approved by the Cornell University Animal Care and Use Committee, Ithaca, USA, (Protocol number 2009–0120). In brief, 12 Holstein heifer calves were randomly assigned at birth to one of two treatments. Calves assigned to the enhanced group were fed 1.26 Megajoules (MJ) of metabolizable energy (ME) per kg BW^0.75^ in three daily feedings. The supply of milk replacer (MR) was adjusted weekly according to changes in BW. Daily amounts of energy supply to each calf increased from 17.58 to 35.17 MJ per day. Calves in the restricted group were fed 11.72 MJ of ME per day in two daily feedings throughout the study, reflecting traditional industry standards. From day 18 onwards, calves were also offered a commercially available concentrate. All calves had access to water and were housed in individual hutches. Information on the composition of milk replacer and concentrate are provided in S1 Table.

### Tissue sample collection and preservation

At 54 days of age, calves were fed at 0700h, weighed and transported to the Cornell University abattoir (Ithaca, NY). Calves were harvested approximately 4h after feeding by stunning with a captive bolt, followed directly by exsanguination. Immediately after exsanguination, a representative sample of omental fat was placed in foil bags and snapped frozen on liquid nitrogen. Samples were then transferred into a -80°C freezer until the gene expression profiling analyses were conducted.

### RNA isolation

Total RNA was prepared by homogenizing the frozen tissues in TRIzol reagent (ThermoFisher Scientific) using an Ultra-Turrax homogenizer (IKA laboratory technology, Staufen, Germany), and purified using Qiagen RNeasy columns (Qiagen, Holden, Germany). RNA integrity was checked on chip analysis (Agilent 2100 Bioanalyzer, Agilent Technologies, Amsterdam, The Netherlands) according to the manufacturer's instructions. RNA was judged as suitable for array hybridization only if samples exhibited intact bands corresponding to the 18S and 28S ribosomal RNA subunits, and displayed no chromosomal peaks or RNA degradation products (RNA Integrity Number > 7.0).

### Affymetrix GeneChip oligoarray hybridization, quality control, and analyses

Total RNA (100 ng) was used for whole transcript cDNA synthesis and terminal labelling using the Ambion WT expression Kit (Life Technologies, Bleiswijk, The Netherlands). Samples were analysed on Affymetrix Bovine Gene 1.1 ST arrays (Affymetrix, Santa Clara, CA). Hybridization, washing, staining and scanning of the arrays were performed automatically on an Affymetrix GeneTitan instrument, according to the manufacturer's instructions. Packages from the Bioconductor project [19], integrated in an online pipeline [20], were used to analyse the array data. Quality of the arrays was determined as described in the S 1 File. Because of insufficient quality of RNA, 2 arrays (1 from each group) were excluded from the final gene expression analysis.

The 553,263 probes on the Bovine Gene ST 1.1 array were redefined utilizing current genome information. In this study, probes were reorganized based on the gene definitions as available in the NCBI database (Bos taurus NCBI Build 6.1; custom CDF version 22), which represented 20,526 unique genes. Normalized expression estimates were obtained from the raw intensity values using the robust multiarray analysis (RMA) preprocessing algorithm available in the library ‘affyPLM’ using default settings [21].

Differentially expressed probe sets were identified using linear models, applying moderated t-statistics that implemented intensity-based empirical Bayes regularization of standard errors (library ‘limma’). The moderated t-test statistic has the same interpretation as an ordinary t-test statistic, except that the standard errors have been moderated across genes (i.e. shrunk to a common value, using a Bayesian model [22,23]). P-values were corrected for multiple testing using a false discovery rate (FDR) method proposed by Storey et al. [24]. Probe sets that satisfied the criterion of FDR < 10% (q-value < 0.10) and p-value < 0.001 were considered to be significantly regulated.

### Bioinformatics analysis

A dataset containing 20,526 gene IDs with respective fold change and p-values was uploaded into the Ingenuity Pathway Analysis software (IPA). Core Analysis was performed using default settings with a p < 0.001 and FDR < 0.1 cut-off, which yielded a total 353 mapped genes ready for analysis.

The Downstream Effects Analysis was used to determine which Molecular and Cellular Functions were most relevant to the DE genes dataset (p < 0.05, B-H correction) and which functions were significantly predicted as decreased or increased (z-scores ≤−2 or ≥+2, respectively).

Upstream Regulator Analysis was used to predict which endogenous regulator genes (e.g. transcriptional regulators, cytokines, ligand dependant nuclear receptors, G-protein coupled receptors, growth factors, kinases, transmembrane receptors, and others) control the expression of downstream DE genes by measuring an overlap p-value (p < 0.05) with Fisher’s Exact test. Their predicted activation/inhibition state (z-scores ≤−2 or ≥2) was also verified based on the collective directional expression of their target genes. Pathway Designer tool was used to generate network illustrations derived from Downstream Effects Analysis (Fatty Acid Metabolism and Mitosis).

The Regulator Effects Analysis (customized settings: max. 1 regulator and 1 function per network generated) was used to determine how the predicted active/inhibited Upstream Regulators impacted downstream DE genes and eventually sub functions. The Upstream Regulators, sub functions and their predicted states were used to generate a customized network to directly link Upstream Regulators states with affected functions in the cellular context. Data visualization was created using the Pathway Designer tool.

## Results

### Differentially expressed genes

When remapping the probes to the **BOS TAURUS genome assembly**, the expression of a total of 19,968 genes was detected (miRNA genes excluded). Expression varied widely among genes due to treatments from very stable genes with expression fold changes (FC) lower than 1.001 up to genes with FC > 13 such as lipocalin-1 and 3-hydroxybutyrate dehydrogenase 1. However, for most genes (94.48%) expression varied less than 50% (FC < +1.5 or FC > -1.5) **(Table 1)**. Still, 1.98% of the genes (396) were considered differentially expressed, satisfying a cut-off level of p-value < 0.001 and FDR < 0.1.

**Table 1:** Transcriptome level alterations in adipose tissue of pre-weaned calves under restricted or enhanced feeding regimes.

	Moderated T-test on gene expression between feeding regimes				
Expression Fold Change*	p > 0.05	p < 0.05	p < 0.01	p < 0.001	Total
**1.0–1.1**	8272	0	0	0	8272 (41.43%)
**1.1–1.2**	5272	177	1	0	5449 (27.29%)
**1.2–1.3**	1915	1074	157	1	2989 (14.97%)
**1.3–1.4**	481	980	366	28	1461 (7.32%)
**1.4–1.5**	160	535	318	67	695 (3.48%)
**1.5–2.0**	142	747	528	200	889 (4.45%)
**>2.0**	23	190	165	100	213 (1.07%)
**Total**	16265 (81.45%)	3703 (18.54%)	1535 (7.69%)	396 (1.98%)	19968 (100%)

*Expression fold change categories include both genes up and down-regulated

### Functional analysis of DE genes between restricted and enhanced diet treatments

**Fig 1:**
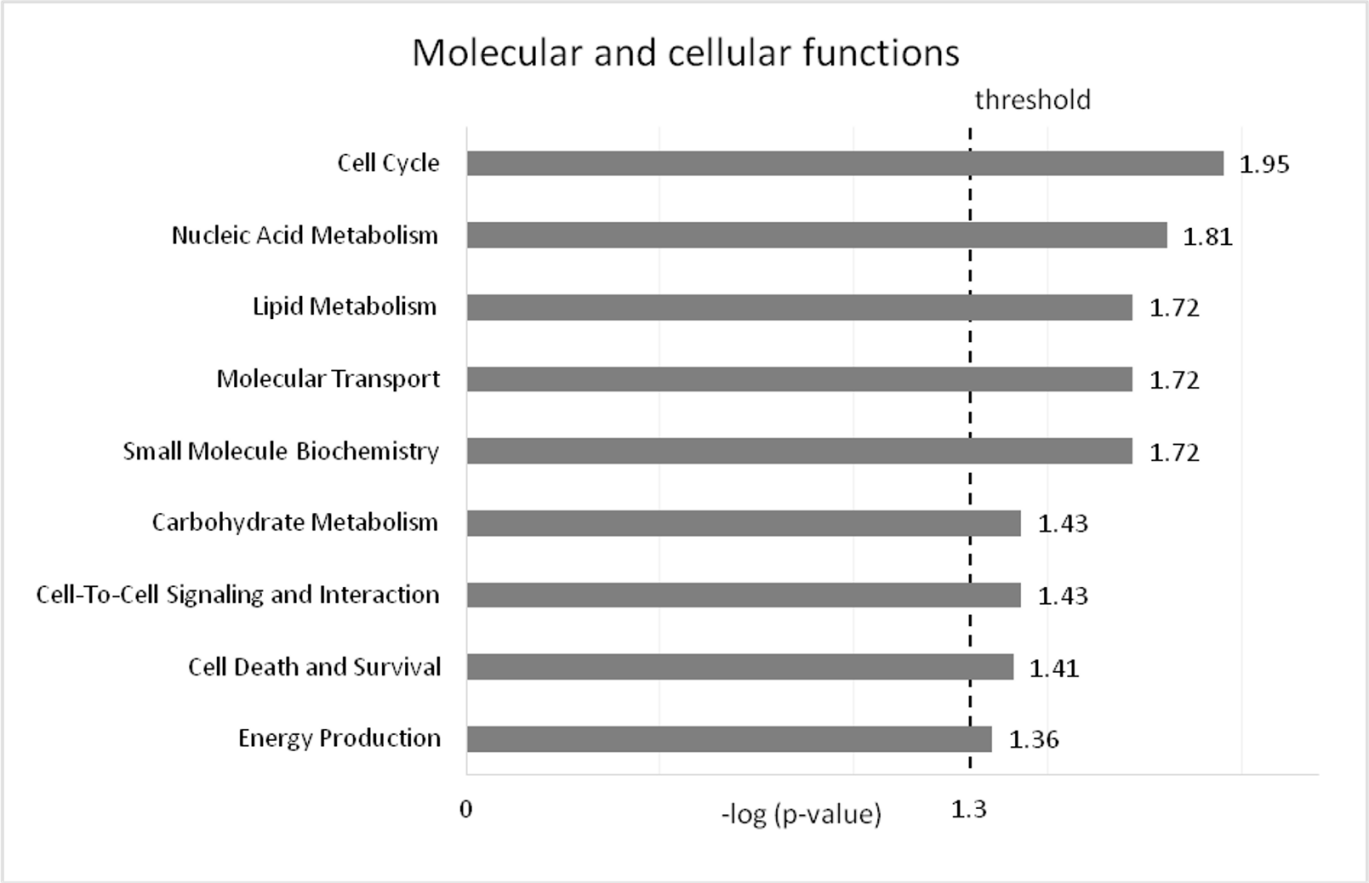
Molecular and cellular functions in DE genes in adipose tissue. The likelihood of the association between the genes in the dataset and a biological function is represented as–log (p-value), with larger bars being more significant than shorter bars. The vertical line indicates the cut-off for significance (p-value of 0.05).

A total of 353 genes out of 396 DE genes (p < 0.001, FDR < 0.1) identified in this study were successfully mapped to the Ingenuity Knowledge Base (IPA). Of these, 220 were up-regulated and 133 down-regulated. Based on this set of genes, IPA Core Analysis revealed that genes differentially expressed due to dietary treatment were involved (p<0.05) in nine Molecular and Cellular Functions **(Fig 1)**. The analysis showed how DE genes are involved in a variety of biological processes. Their roles included several aspects of adipose cell metabolism, with the top functions ranging from Cell Cycle, which consists mainly of DNA replication and cell division, to Lipid Metabolism, considered the prime function of adipose tissue. The amount of genes involved in each function varied considerably, with the most in Cell Death and Survival (118 genes) and the least in Energy Production (14 genes).

**Fig 2:**
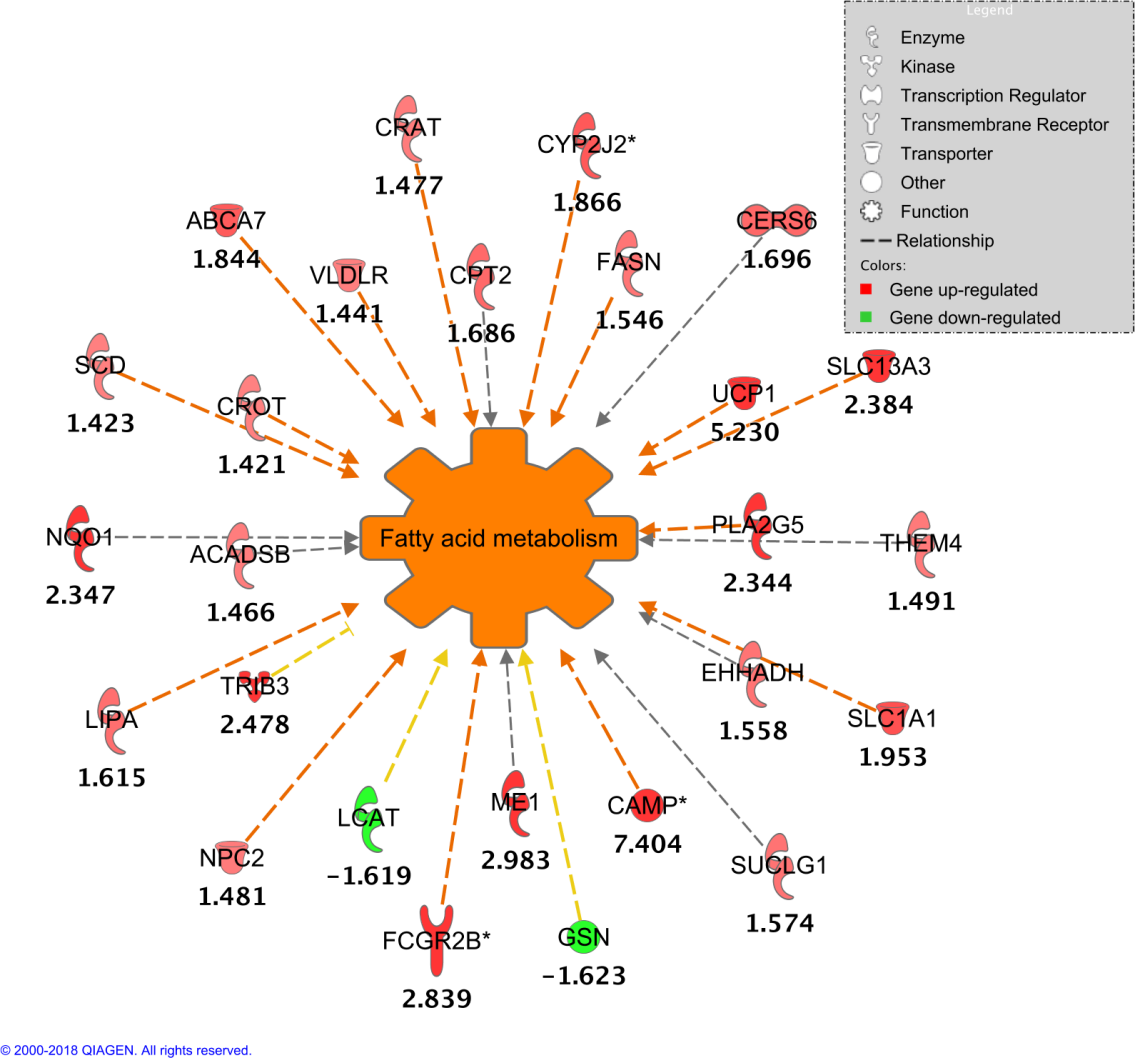
Effects of DE genes in Fatty Acid Metabolism. Values under gene symbols represent direction expression of DE genes (fold change). Relationship lines in orange indicate that genes showed a directional expression compatible with an increased function. Yellow lines indicate that the gene expression is compatible with a decreased function. Gray lines mean that the genes are involved in this function, but literature results in Ingenuity Knowledge Base do not indicate whether they increase or decrease Fatty Acid Metabolism.

Data was further processed through Downstream Effects Analysis in order to predict which biological processes were increased or decreased based on the directional expression of the involved DE genes. A total of eight predictions of sub functions activation states were statistically significant (Z-score > +2). They derived from the following main Molecular and Cellular Functions categories: Lipid Metabolism, Small Molecule Biochemistry, Cellular Transport, Cell Cycle, and Cellular Assembly and Organization (**Table 2**). Z-scores for sub functions were all positive (Z-score > +2), indicating functional increase. Fatty Acid Metabolism was the only sub function from Lipid Metabolism category that was significantly predicted to be increased and it was the process with the overall strongest activation prediction (Z-score = + 2.605) with a total of 26 DE genes involved. The directional expression of most of these 26 genes indicated that calves under the enhanced nutrient supply had a significant increase in Fatty Acid Metabolism function (**Fig 2**). This Downstream Effect Analysis result (Fatty Acid Metabolism increase) is based on the Ingenuity knowledge Base that indicated that out of these 26 genes, 15 genes showed a directional expression compatible with an increased function (ABCA7, CAMP, CRAT, CROT, CYP2J2, FASN, FCGR2B, LIPA, NPC2, UCP1, PLA2G5, SCD, SLC13A3, SLC1A1, and VLDLR), 3 genes had expressions compatible with a decreased function (GSN, LCAT, and TRIB3) and 8 gene are involved in this function (ACADSB, CERS6, CPT2, EHHADH, ME1, NQO1, SUCLG1, and THEM4) but literature results, in Ingenuity Knowledge Base, are not consistent whether they increase or decrease it. (**Fig 2**).

**Table 2:** Biological functions predicted to be activated by Downstream Effects Analysis and DE genes involved.

Categories	Functions Annotation	p-Value	Predicted Activation State	Activation z-score	Molecules	# Molecules
**Lipid Metabolism, Small Molecule Biochemistry**	Fatty Acid Metabolism	0.00073	Increased	2.605	ABCA7,ACADSB,CAMP,CERS6,CPT2,CRAT,CROT,CYP2J2,EHHADH,FASN,FCGR2B,GSN,LCAT,LIPA,ME1,NPC2,NQO1,PLA2G5,SCD,SLC13A3,SLC1A1,SUCLG1,THEM4,TRIB3,UCP1,VLDLR	26
**Molecular Transport**	Transport of Carboxylic Acid	0.00075	Increased	2.429	CPT2,CRAT,CROT,SLC13A3,SLC16A7,Slc25a1,SLC26A8,UCP1	8
**Cell Cycle**	Interphase	0.00027	Increased	2.361	AATF,ABL1,ABRAXAS1,ATF5,BUB1B,CCNB1,CCNE1,CCNE2,CDC20,CDK1,CDS1,EDN3,ETS1,FASN,FGF1,GPI,IGFBP5,IL6R,ITGA6,KAT5,KIF11,MCM10,MET,PCBP4,PCLAF,RBM14,RHOU,RRM2B,TIMP2,ZBTB10	30
**Cellular Assembly and Organization**	Development of Cytoplasm	0.0136	Increased	2.261	AATF,ABL1,BAIAP2L1,CAMP,CENPF,DLGAP5,FGF1,GNA11,GPI,GSN,INPP5B,INPPL1,MET,MSRB2,MYADM,NPR1,NUSAP1,RANBP9,S100A10	19
**Cell Cycle**	Cell cycle Progression	0.00021	Increased	2.212	AATF,ABL1,ANLN,BUB1B,CCNB1,CCNE1,CCNE2,CDC20,CDK1,CENPA,CENPE,CENPF,CKAP2,DLGAP5,EDN3,ETS1,FASN,FCGR2B,FGF1,GPI,IGFBP5,IL6R,KIF11,MET,MKI67,NUSAP1,OGT,PATZ1,PCBP4,PCLAF,PLAGL1,PNN,PRMT1,RHOU,SERPINA5,SON,SSRP1,STAT6,TAF1D,TCERG1,TGFB2,TIMP2,ZBTB10	43
**Cell Cycle**	Entry into Interphase	0.00041	Increased	2.159	ABL1,BUB1B,CCNE1,CDC20,CDK1,FGF1,IL6R,ITGA6,MCM10,MET	10
**Cellular Assembly and Organization**	Formation of Cytoskeleton	0.00517	Increased	2.092	ABL1,BAIAP2L1,CENPF,DLGAP5,FGF1,GNA11,GPI,GSN,INPP5B,INPPL1,MET,MSRB2,MYADM,NPR1,NUSAP1,RANBP9,S100A10	17
**Cell Cycle**	Mitosis	0.00066	Increased	2.011	AATF,ABL1,ANLN,BUB1B,CCNB1,CCNE1,CDC20,CDK1,CENPA,CENPE,CENPF,CKAP2,DLGAP5,EDN3,FGF1,IGFBP5,KIF11,MET,MKI67,NUSAP1,STAT6,TCERG1,TIMP2	23

**Fig 3:**
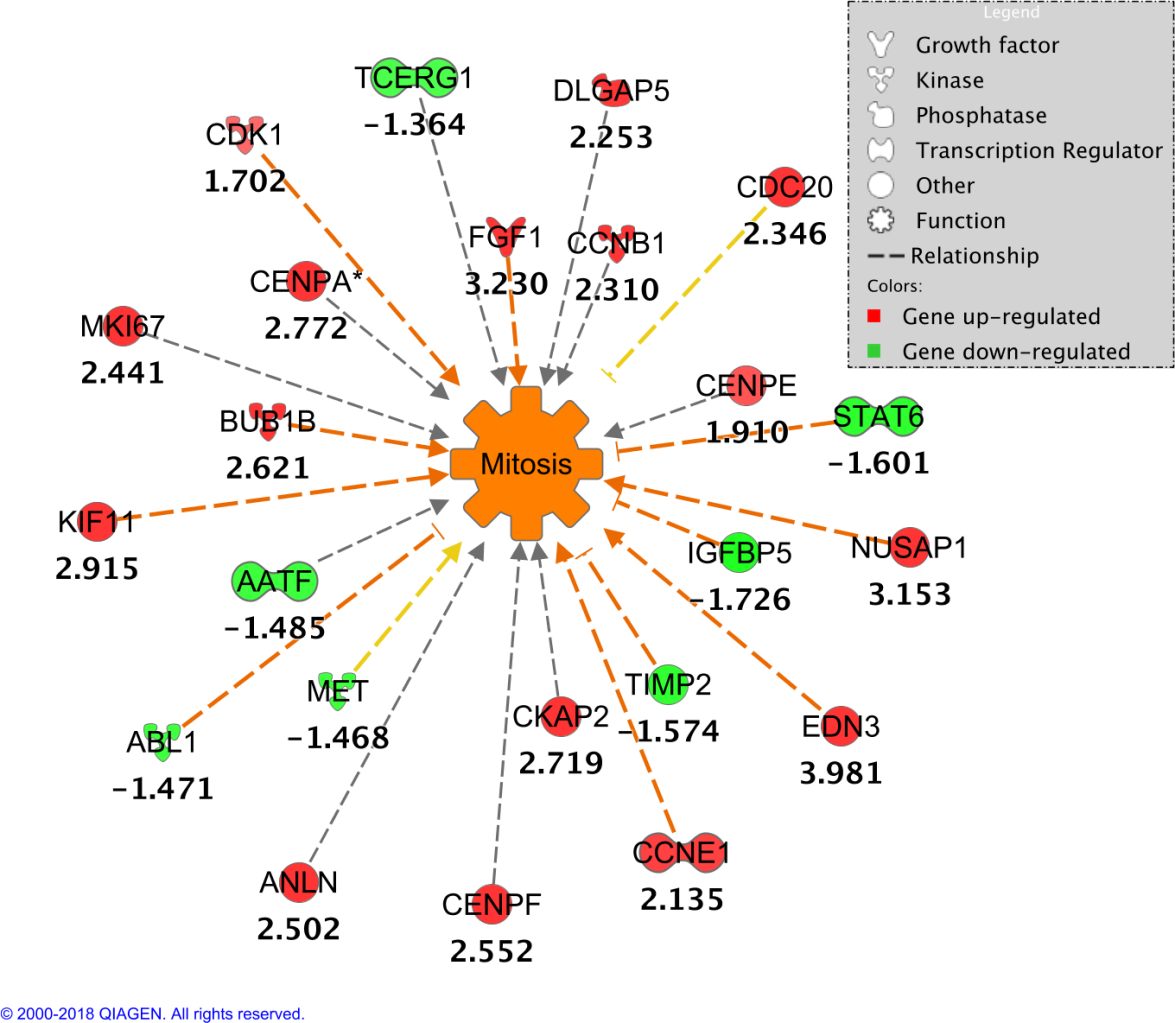
Effects of DE genes in Mitosis. Values under gene symbols represent direction expression of DE genes (fold change). Relationship lines in orange indicate that genes showed a directional expression compatible with an increased function. Yellow lines indicate that the gene expression is compatible with a decreased function. Gray lines mean that the genes are involved in this function, but literature results in Ingenuity Knowledge Base do not indicate whether they increase or decrease Fatty Acid Metabolism.

Cell Cycle was the functional category most represented, with four sub functions significantly predicted to be increased: Interphase (30 DE genes), Cell Cycle Progression (43 DE genes), Entry into Interphase (10 DE genes), and Mitosis (23 DE genes). Ingenuity knowledge Base indicated that out of these 23 DE genes involved in Mitosis, 11 genes showed a directional expression compatible with an increased function (BUB1B, ABL1, CCNE1, KIF11, STAT6, IGFBP5, FGF1, NUSAP1, EDN3, TIMP2, and CDK1), 2 genes (CDC20 and MET) had expressions compatible with a decreased function, and 10 genes (MKI67, CKAP2, DLGAP5, CENPA, CCNB1, AATF, TCERG1, ANLN, CENPF, and CENPE) were involved in this function but literature results, in Ingenuity Knowledge Base, do not indicate whether they increase or decrease Mitosis (**Fig 3**).

### Upstream regulators and effects

Upstream Regulator Analysis identified a total of 346 molecules as potential upstream regulators for the set of 353 DE genes, including transcriptional regulators, cytokines, ligand dependant nuclear receptors, G-protein coupled receptors, growth factors, kinases, transmembrane receptors, and others, but excluding drugs and chemicals. However, the analysis was able to reveal the activation status of only 23 Upstream Regulators based on the directional expression of their controlled DE genes (p-value of overlap < 0.05). Most of them (13 genes) were predicted to be activated (PPARGC1A, CSF2, PPARG, AHR, FOXM1, PTGER2, FGF21, KLF15, RABL6, E2F3, MED1, PPARA, and SMOC2) with a Z-score higher than +2, while 10 were predicted to be inhibited (RICTOR, MAP4K4, INSIG1, LGR4, LGR5, SMARCE1, SMARCB1, NUPR1, TCF3, and BNIP3L) with a Z-score lower than -2 (**Table 3**). This set of Upstream Regulators accounted for the molecular regulation of 108 out of 353 DE genes identified in this study. These transcriptional factors varied on how they influence DE genes regulation, from 4 DE genes by SMOC2 up to 22 DE genes by PPARG (**Table 3**).

**Table 3:** Predicted activation status of upstream regulators that control differentially expressed genes and their targets.

Upstream Regulator	Predicted Activation State	Activation z-score	Expr Fold Change	p-value of overlap	# genes	Target genes in DE dataset (108 out of 353)
**PPARGC1A**^**1**^	Activated	3.517	1.576	0.000183	13(**↑**13/**↓**0)	↑: BDH1, FASN, IMPA1, LIPA, ME1, NDUFS1, NDUFS8, OXCT1, PRDX3, SCD, SIRT3, TRIB3, UCP1
**CSF2**^**2**^	Activated	3.299	1.138	0.00783	15(**↑**13/**↓**2)	↑: ANLN, BUB1B, CCNB1, CDC20, CDK1, CENPE, FCGR2B, HJURP, KIF11, MKI67, MNS1, NUSAP1, RRM2B. ↓: ILF3, MET
**PPARG**^**3**^	Activated	3.193	-1.059	0.00000872	22(**↑**18/**↓**4)	↑: ACADS, BDH1, CPT2, CRAT, DHRS3, EHHADH, FASN, FBP2, FGF1, LHPP, ME1, MKI67, SCD, Slc25a1, TKT, TRIB3, UCP1, VLDLR. ↓: ETS1, IGFBP5, RARRES2, SLC44A1
**AHR**^**3**^	Activated	2.873	-1.056	0.000482	15(**↑**10/**↓**5)	↑: ABCB6, ALDH2, CCNB1, CCNE1, CDK1, ITGA7, NQO1, SCD, SLC16A10, SLC1A1. ↓: COLQ, EMILIN2, ITGA6TGFB2, TIMP2
**FOXM1**^**1**^	Activated	2.771	1.385	0.00000497	11(**↑**10/**↓**1)	↑: BUB1B, CCNB1, CCNE1, CCNE2, CDC20, CDK1, CENPA, CENPE, CENPF, MKI67. ↓: MET
**PTGER2**^**4**^	Activated	2.646	1.072	0.00164	7(↑6/1↓)	↑: BUB1B, CENPE, CENPF, KIF11, MKI67, NUSAP1. ↓: TIMP2
**FGF21**^**5**^	Activated	2.398	-1.091	0.0000838	6(6↑/0↓)	↑: BDH1, COX7A1, FASN, ME1, Slc25a1, UCP1
**KLF15**^**1**^	Activated	2.345	1.129	0.0000736	6(**↑**6/**↓**0)	↑: ACADS, CPT2, EHHADH, FASN, NPHS2, SCD
**RABL6**^**6**^	Activated	2.236	-1.006	0.00408	5(5↑/0↓)	↑: BUB1B, CCNB1, CCNE2, CENPF, MCM10
**E2F3**^**1**^	Activated	2.236	1.178	0.0259	7(**↑**7/**↓**0)	↑: BAIAP2L1, CCNB1, CCNE1, CDK1, MCM10, ORC1, PCLAF
**MED1**^**1**^	Activated	2.158	-1.201	0.00091	9(**↑**8/**↓**1)	↑: CCNB1, CCNE1, CDC20, CDK1, CENPA, EHHADH, FASN, UCP1. ↓: MET
**PPARA**^**3**^	Activated	2.13	1.351	0.0000608	20(**↑**16/**↓**4)	↑: ACADS, ALDH2, CCNB1, CCNE1, CDK1, CPT2, CROT, EHHADH, FASN, G6PD, HPD, MKI67, SCD, SORD, UCP1, UQCRC1. ↓: C2, IGFBP5, RBP7, SRM
**SMOC2**^**6**^	Activated	2	1.167	0.0000133	4(4↑/0↓)	↑: CCNB1, CDK1, CENPF, MCM10
**RICTOR**^**6**^	Inhibited	-2.138	-1.12	0.000119	14(11↑/3↓)	↑: ATP6V1E1, COX17, Cox6c, COX7A1, LHPP, NDUFA10, NDUFS1, NDUFS2, NDUFS8, UQCR10, UQCRC1. ↓: RPL14, RPL35A, RPL8
**MAP4K4**^**7**^	Inhibited	-2.157	-1.089	0.000693	8(7↑/1↓)	↑: ACADS, FASN, NDUFS1, NDUFS8, SCD, SUCLG1, UQCRC1. ↓: PIGO
**INSIG1**^**6**^	Inhibited	-2.158	2.372	0.0254	5(5↑/0↓)	↑: FASN, G6PD, LIPA, SCD, TM7SF2
**LGR4**^**8**^	Inhibited	-2.236	-1.068	0.000238	5(5↑/0↓)	↑: Gulo, LHPP, OAT, SLC13A3, SLC16A10
**LGR5**^**8**^	Inhibited	-2.236	1.084	0.000018	5(5↑/0↓)	↑: Gulo, LHPP, OAT, SLC13A3, SLC16A10
**SMARCE1**^**1**^	Inhibited	-2.236	-1.231	0.000000775	6(**↑**6/**↓**0)	↑: CCNB1, CCNE1, CDK1, CENPE, CENPF, NPC2
**SMARCB1**^**1**^	Inhibited	-2.236	-1.289	0.0207	7(**↑**6/**↓**1)	↑: CCNE1, CDK1, CENPA, CENPE, KIF11, MCM10. ↓: GSN
**NUPR1**^**1**^	Inhibited	-2.357	1.518	0.0016	18(**↑**14/**↓**4)	↑: BUB1B, CROT, FGF1, HIST1H2AI, HJURP, KIF11, MCM10, MKI67, MTFMT, RIMKLA, SHCBP1, SLC16A10, TMEM19, TRIB3. ↓: ETS1, IL6R, KAT5, RBM14
**TCF3**^**1**^	Inhibited	-2.449	-1.13	0.00841	12(**↑**9/**↓**3)	↑: ANLN, CCNB1, CCNE1, CCNE2, CDC20, CDK1, KIF11, MKI67, NUSAP1. ↓: AATF, DAB2IP, RASGRP2
**BNIP3L**^**6**^	Inhibited	-2.63	1.044	0.0000291	7(↑5/↓2)	↑: CCNE2, CENPE, CENPF, CKAP2, KIF11. ↓: GSN, TIMP2

1.Transcriptional regulator

2.Cytokine

3.Ligand dependant nuclear receptor

4.G-protein coupled receptor

5.Growth factor

6.Other

7.Kinase

8.Transmembrane receptor.

↑ indicates genes up-regulated. ↓ indicates genes down-regulated. Only upstream regulators with an overlap *p*-value < 0.05 were displayed.

**Fig 4:**
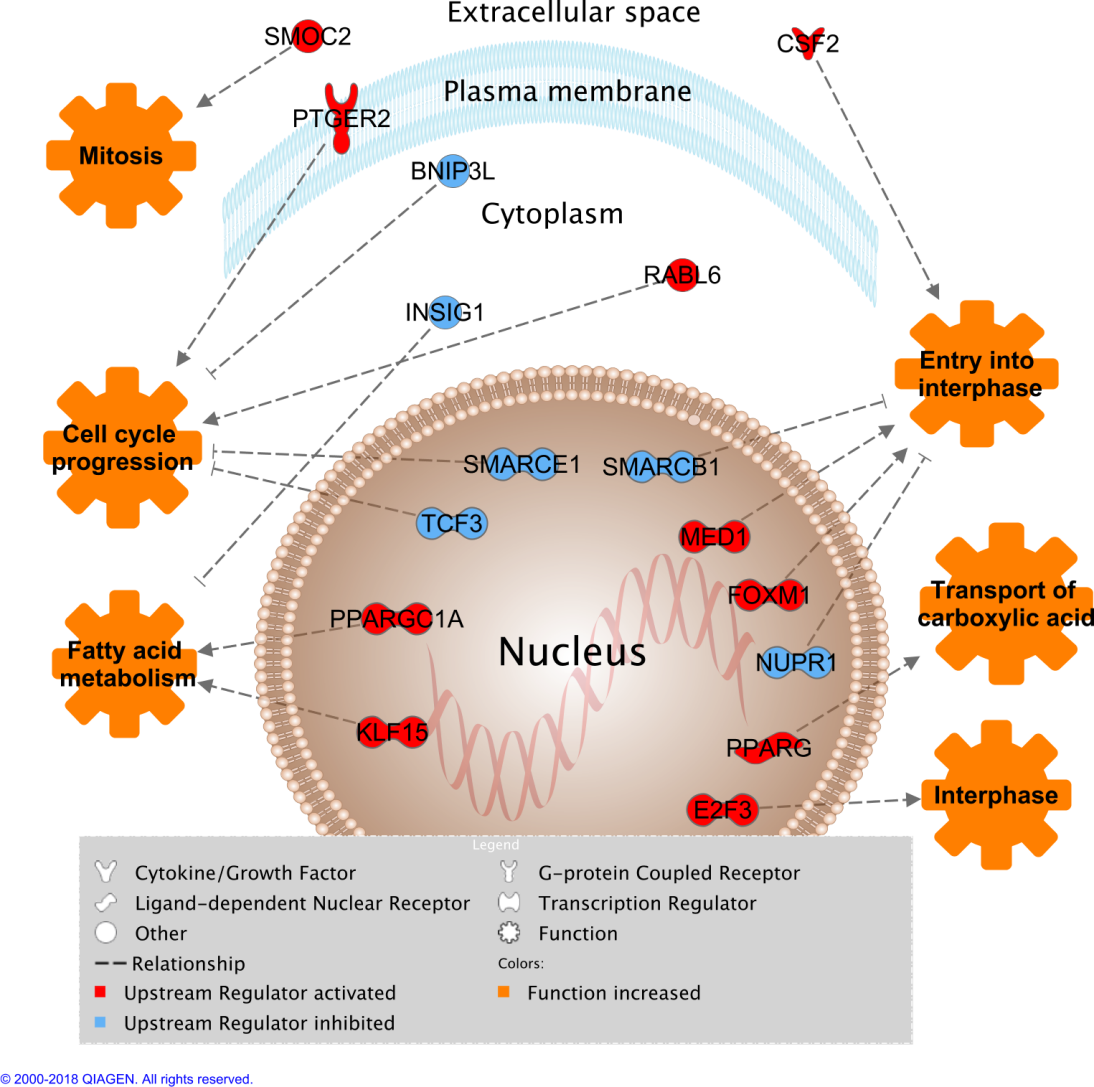
Predicted impact of upstream regulators controlling DE genes on adipose tissue biological functions. Only upstream regulators with overlap p-value < 0.05 and Z-score > +2 or < -2 are displayed.

A total of 17 upstream regulators were further analyzed through IPA’s Regulator Effects feature in order to reveal not only what genes they are likely to control, but ultimately what functions the identified Upstream Regulators are affecting (**Fig 4**). By combining the activation status of the Upstream Regulators with the directional expression change of the genes they regulate and Ingenuity Knowledge Base information, this analysis provided evidence indicating that TCF3, SMARCE1, RABL6, BNIPL3, and PTGER2 were predicted to be involved in the regulating of Cell Cycle Progression. FOXM1, MED1, SMARCB1, NUPR1, and CSF2 were involved in Entry into Interphase. INSIG1, KLF15, and PPARGC1A were regulating Fatty Acid Metabolism. E2F3 was regulating Interphase related functions. SMOC2 was regulating Mitosis. PPARG was involved in Transport of Carboxylic Acid. AHR was predicted to be involved in Fibrosis of Liver aspects, however, this result was not included as samples were derived from adipose tissue. PPARA did not appear in any result of Regulator Effects feature (**Fig 4**).

## Discussion

Adipose tissue is a dynamic organ and plays a crucial role in energy balance. Several factors have been reported to impact the molecular regulation of adipose tissue in bovine such as age [25], depot location [17] and diet [16]. However, it is not clear how early life dietary manipulation can affect adipose tissue transcriptome and function. In the present study, two feeding regimes were used, one simulating a restricted nutrient supply, a practice that is still used by part of the dairy industry, while an alternative feeding regimen provided an enhanced nutrient supply (higher amount of milk replacer). Calves in the first group were heavily restricted in their caloric intake over maintenance–based on NRC 2001 [26]–compared with calves under the enhanced diet (3.77 MJ/day *vs*. 15.91 MJ/day, respectively).

As previously reported [18], calves in the enhanced nutrient supply group had higher total milk replacer intakes than restricted calves (69.5 and 32.6 kg dry matter, respectively) and were larger at harvest at 54±2 days old (83.2 and 61.0 kg, respectively). The large difference in nutrient supply affected not only the overall body development of calves, but also increased (p<0.01) mammary gland and liver growth, as a percentage of body weight [18].

In this manuscript we further show that an enhanced nutrient supply also alters adipose tissue molecular regulation. The dietary treatment altered the adipose transcriptome molecular regulation as gene expression changed by more than 1.5 FC for more than a thousand genes (**Table 1**). Adipose tissue seems to be highly responsive to dietary treatments. Transcriptome data from the same animals indicate that adipose tissue genes were more affected by the treatment compared to muscle and bone marrow[27]. Also, in a study in which pre-weaning calves were fed a restricted vs. an ad libitum milk replacer supply, transcriptome changes in the jejunum were more discrete than the changes observed in the present study, as 244 genes were differentially expressed (p-value < 0.001) [28] while at the same cut-off adipose tissue in our study had 396 genes differentially expressed. However, samples in our experiment were collected at 54 days, while jejunum samples were collected only at 80 days of age, four weeks after our experimental diets.

White adipocytes are considered the main cellular type in adipose tissue; however this organ is also composed of other cells such as preadipocytes, brown adipocytes, beige adipocytes, macrophages, endothelial cells and stem cells [29]. Therefore, the results of the functional analysis on DE genes in this study reflect not only white adipocyte cells, but also the biology of the whole organ. Besides, each adipose tissue depot has a distinctive physiology and specific molecular regulation [17,30]. Omental fat, for example, is considered a visceral depot. However; unlike other major fat depots (e.g. subcutaneous and perirenal fat) it contains lymphoid aggregates, known as milky spots, which allow omental fat tissue to play a role in peritoneal immunity [31]. The functional analysis revealed DE genes in omental fat where involved in a variety of cellular processes (**Fig 1**). Cell Cycle appeared as the most significant function for the DE genes set. This function is related to the process of cell reproduction. Adipose tissue development happens through two distinct processes: hypertrophy (increase in cell size) and hyperplasia (increase in cell number) [32]. The later process corresponds to the enriched Cell Cycle function. Not surprisingly, Lipid Metabolism appeared as one of the top functions, as adipose tissue is the premier body location for lipid processing.

Downstream Effects Analysis (**Table 2**) further revealed the predicted activation state of sub functions based on the directional expression of DE genes involved. Fatty Acid Metabolism, which belongs to the Lipid Metabolism category, was the function with the highest predicted activation state (z-score). This finding is based on the directional expression of the 26 DE genes involved (**Fig 2**), which indicates that adipose tissue of calves under an enhanced nutrient supply are processing fatty acids at a higher rate compared to restricted animals. Another study with similar treatments reported that the gene network ‘energy production, lipid metabolism and small molecule biochemistry network’ was enriched in subcutaneous adipose tissue of calves (126 day old) fed an enhanced diet [33]. Older animals (197-day-old Angus steers) under an enhanced nutrient supply (high concentrate diet vs. pasture) also displayed altered transcriptome expression in subcutaneous adipose tissue (1113 DE genes), indicating increased lipid metabolism functions, including Fatty Acid Metabolism [34].

A higher nutrient intake allows adipocytes to increase fat stores by either an increased uptake of free fatty acids or de novo lipogenesis [32,35]. This condition provides the requirements for an increased adipose tissue expansion. Downstream Effects Analysis (**Table 2**) further showed that four sub functions belonging to Cell Cycle category were predicted to be increased: Entry into Interphase, Interphase, Cell Cycle Progression, and Mitosis. These results indicate that omental fat in the calves under the enhanced nutrient supply experienced an increased tissue expansion through hyperplasia. However, hypertrophy might have also contributed to adipose tissue development since the sub functions Development of Cytoplasm and Formation of Cytoskeleton (Cellular Assembly and Organization category) were predicted to be increased. These findings are evidence that early life nutrient supply influences the genetic regulation of hyperplastic and hypertrophic processes in calves’ adipose tissue. Our findings are supported by another study investigating low vs. high plane of nutrition in calves’ subcutaneous adipose tissue histology and transcriptome [33]. Histology results indicated that at 126 days of age, calves in a low plane of nutrition displayed less adiposity (lower quantity of adipocytes, smaller adipocyte diameter and more preadipocytes) when compared to calves in a high plane of nutrition [33].

In another study, pre-weaned Friesian calves fed an enhanced vs. restricted nutrient supply displayed increased adipose cell hypertrophy, but hyperplasia did not differ at 95 days; however after weaning treatment diets were paired until slaughter at 553 days where no differences in adipose cellularity were observed [36], which might represent a compensatory developmental mechanism.

The regulation of gene expression in bovine adipose tissue is complex. There is a multilayered system with different control levels to ensure that gene expression is fine tuned, including epigenetic modifications (e.g DNA methylation and histone modifications) [1], transcriptional regulation (e.g transcriptional factors) [37] and post-transcriptional regulation (microRNAs) [38]. Transcriptional factors are major players in regulating the expression of downstream genes. However, their expression (mRNA level) is not a safe indication of its activity since their activity is mainly controlled by a post-translational modification such as protein phosphorylation or ligand binding [39]. The Upstream Regulator Analysis identified a high amount of potential upstream regulators for the DE genes, including not only transcriptional factors, but also cytokines, G-protein coupled receptors and others (n = 436). However, only 23 Upstream Regulators obtained a significant prediction of activation or inhibition considering the expression profile of the DE genes they control (**Table 3**). Even though none of these 23 upstream regulators were differentially expressed, they are likely to orchestrate a significant part of the effects of the enhanced nutrient supply on adipose tissue regulation, since 108 DE genes were predicted to be under their control.

The combination of customized Regulator Effects analysis results with a Network construction with exclusion of regulated genes provided a simplified view of the impact of the dietary intervention on Upstream Regulators and consequentially on Molecular and Cellular functions of adipose cells (**Fig 4**). The dietary effects can be grouped mainly into Lipid Metabolism and Cell Cycle functions. PPARG, PPARGC1A, KLF15 and INSIG1 are known to affect not only Lipid Metabolism but also adipogenesis. PPARG is the master regulator of adipogenesis and regulates several aspects of Lipid Metabolism [37,40], such as the Transport of Carboxylic Acid, which comprise fatty acids. This transcription factor has binding sites in most lipid metabolism genes [41]. Its predicted activated state is an indicator that adipose tissue is developing and expanding through adipogenesis and lipogenesis. The transcriptional factor with the highest activation score was another lipid metabolism gene, PPARGC1A, which is a co-activator of PPARG and is involved in Fatty Acid Metabolism regulation as well as in the activation of adaptive thermogenesis [42]. KLF15 was also a transcription factor predicted to be active that is involved in adipogenesis and Lipid Metabolism aspects. The deletion of this gene in adipose tissue was shown to decrease adiposity and mechanistic studies indicate it regulates lipogenesis genes and inhibits lipolysis genes in adipocytes [43]. INSIG1 was the only Upstream Regulator involved in Lipid Metabolism aspects that was predicted to be inhibited. This gene was reported to inhibit lipogenesis and preadipocyte differentiation [44].

The other Upstream Regulators (BNIPL3, CSF2, E2F3, FOXM1, MED1, NUPR1, PTGER2, RABL6, SMARCB1, SMARCE1, SMOC2, and TCF3) were affecting functions related to Cell Cycle (Entry in Interphase, Interphase, Cell Cycle Progression, and Mitosis) (**Fig 4**). Three transcriptional factors predicted as active (MED1, FOXM1, and E2F3) are known to be involved in the regulation of Interphase steps. E2F3 activates genes that control the rate of proliferation of cells [45] and its immunodepletion inhibits the induction of the S phase (stage of chromosomes replication) [46]. Similarly, FOXM1 induces the expression of several genes that regulate G1/S transition and DNA replication [47], and moreover, influences G2/M transition and Mitosis [48]. Other transcriptional factors (NUPR1, SMARCE1, SMARCEB1, and TCF3) affecting Cell Cycle functions were predicted as inhibited. TCF3, for example, is a transcriptional factor that inhibits the Wnt target genes [49], and in somatic cells, the activation of Wnt pathway induce cell proliferation [50,51]. In addition to transcriptional factors, other categories of Upstream Regulators located outside the nucleus have also been reported to stimulate Cell Cycle Progression such as RABL6 in cytoplasm [52] and PTGER2 in the plasma membrane [53].

The results of this study revealed that an early life enhanced diet changes adipose tissue physiology by increasing the metabolism of fatty acids and cell proliferation. Fat mass of calves from enhanced diet was likely to be increased as Leptin expression was higher (**S2 Table**). Another study also found that calves supplied with an enhanced diet display increased Letpin levels compared to calves in a restricted diet [33] and it is known that Leptin expression correlates positively with fat mass [54,55] and. However, the expression of Tumor Necrosis Factor α (TNF) and Interleukin 6 (IL6), which are pro-inflammatory cytokines [56,57], did not differ (S2 Table), indicating that although the amount of adipose tissue of calves under an enhanced diet was higher, this organ was healthy, not inducing inflammation and its consequences. Underfeeding or overfeeding animals is a concern. A sustained energy imbalance with deficient or excessive energy intake compared to energy expenditure, may lead to abnormal amounts of adipose tissue and health problems. Excessive fat mass can lead to local and systemic health problems such as hypoxia in adipose tissue cells [58], inflammation and insulin resistance [59,60]. In adult cows it may also lead to fertility [5] and metabolic problems during lactation [6]. Conversely, a restricted calorie intake in young animals may lead not only to a reduced fat mass, but also to a delayed immune system maturation [61] and even a lower milk yield at adult life [3].

An interesting finding that might represent another functional difference between calves treated with an enhanced nutrient supply was the increased expression of Uncoupling Protein 1 (UCP1) (S2 Table). This mitochondrial membrane protein is expressed only in brown and beige adipocytes [62,63], which are likely to be part of the omental adipose tissue of calves in this experiment. Beige adipocytes are inducible and widely distributed in various white adipose tissues such as subcutaneous, epididymal and mesenteric depots [64]. Like brown adipocytes, beige adipocytes have been shown to dissipate chemical energy in the form of heat and play an important role in non-shivering thermoregulation and glucose/insulin sensitivity [63,65]. Uncoupling Protein 1 expression is controlled by the transcriptional factor PPARGC1A, which was predicted to be activated. PPARGC1A induces the adaptive thermogenesis program responsible for turning chemical energy into heat (non-shivering thermogenesis) [40,66] and UCP1 is the key player in this processes [67]. However, it is not clear whether this finding indicates that calves fed an enhanced diet developed an increased thermoregulation control through non-shivering thermogenesis or if this gene expression response was a diet induced thermogenesis phenomenon in which the purpose was not thermoregulation, but to regulate the amount of lipid stores through heat generation to control body weight [68].

In conclusion, adipose tissue of pre-weaning female calves is developmentally and metabolically responsive to early life nutrient supply (enhanced vs. restricted). However, it is still not clear if it imprints adipose physiology and under what conditions it positively or negatively impact future animal performance. Additional studies considering both growing and productive stages will lead to a better understanding on how neonatal nutrient supply alters long term adipose function and how that relates to animal performance.

## Supporting information

S1 Table Milk replacer and starter grain chemical composition as reported by manufacturer. (DOCX)

S2 Table Overall genes expression. (XLSX)

S1 File Array quality. (DOCX)
