# Supplementary material for: Nutrient supply alters transcriptome regulation in adipose tissue of pre-weaning Holstein calves
Source: PLoS One. 2018 Aug 6;13(8):e0201929. doi: 10.1371/journal.pone.0201929 (PMC6078305; doi:10.1371/journal.pone.0201929)
Supplement: S1 Table — (DOCX) [file pone.0201929.s001.docx]

| **S 1 Table.** Milk replacer and starter grain chemical composition as reported by manufacturer. | | |
| --- | --- | --- |
| **Analysis on dry matter basis** | **Milk replacer^1^** | **Starter grain^2^** |
| Crude protein, % | 28.5 | 22 |
| Crude fat, % | 15 | 4.3 |
| Neutral detergent fiber, % | 0.2 | 33.1 |
| Calcium, % | 1 | 1.6 |
| Phosphorus, % | > 0.6 | 1 |
| Vitamin A, IU/g | > 16.5 | 43.1 |
| Vitamin D3, IU/g | > 5.5 | NA |
| Vitamin E, IU/kg | > 110.3 | 197 |
| Gross energy, MJ/kg | 21.35 | 7.54 |
| ^1^Excelerate, Milk Specialties Inc, Carpentersville, IL.  ^2^Cargill, Inc.  NA = not available | | |
