# Supplementary material for: Nutrient supply alters transcriptome regulation in adipose tissue of pre-weaning Holstein calves
Source: PLoS One. 2018 Aug 6;13(8):e0201929. doi: 10.1371/journal.pone.0201929 (PMC6078305; doi:10.1371/journal.pone.0201929)
Supplement: S1 File — (DOCX) [file pone.0201929.s003.docx]

**S 1 File.** Array Quality

Array quality. Various advanced quality metrics, diagnostic plots, pseudoimages, and classification methods were used to determine the quality of the arrays prior statistical analysis. Briefly, for each array the average background signal, percentage present calls, and scale factors were determined, essentially as described [1]. Two arrays did not pass the guidelines recommended by Affymetrix, as described in the Affymetrix Microarray Suite Users Guide, Version 5.0 (2001). In addition, the library ‘AffyPLM’ was used to fit probe-level linear models that provide parameter estimates for probes and arrays on a probe by probes basis. Two-dimensional pseudoimages of the arrays based on probe-level quantities, namely the weights and residuals computed by fitPLM, were inspected for stains, scratches and other artifacts. Even if present, all artifacts covered less than 5% of the area of the array. Moreover, numerical quality was assessed based on two distributions computed at the probeset level, the normalized unscaled standard error (NUSE) and relative log expression (RLE). The remaining 10 arrays used for further interpretation were observed for all these QC parameters, i.e. arrays were reasonably centered around the median NUSE = 1, and boxplots for RLE had a small spread and were centered at RLE = 0.

Reference

1. Gregory Alvord W, Roayaei JA, Quinones OA, Schneider KT (2007) A microarray analysis for differential gene expression in the soybean genome using Bioconductor and R. Brief Bioinform 8: 415-431.
